# Laparoscopic Versus Robotic Lateral Pelvic Lymph Node Dissection in Locally‐Advanced Rectal Cancer: A Cohort Study Comparing Perioperative Morbidity and Short‐Term Oncological Outcomes

**DOI:** 10.1002/cnr2.70174

**Published:** 2025-03-07

**Authors:** Joseph Mathew, Yogesh Kisan Bansod, Nishant Yadav, Janesh Murugan, Kovvuru Bhaskar Reddy, Mufaddal Kazi, Ashwin DeSouza, Avanish Saklani

**Affiliations:** ^1^ Department of GI Surgical Oncology and Minimal Access Surgery HealthCare Global Enterprises Ltd. (HCG) Bangalore India; ^2^ Division of Colorectal Oncology, Department of Surgical Oncology Tata Memorial Centre Mumbai India; ^3^ Department of Surgical Oncology MPMMCC Tata Memorial Centre Varanasi India

**Keywords:** cancer management, clinical cancer research, clinical outcome, colorectal cancer, surgical oncology, surgical therapy

## Abstract

**Background:**

Robotic surgery has been associated with superior short‐term outcomes in patients undergoing total mesorectal excision (TME) for organ‐confined rectal cancer. However, whether this approach offers an additional benefit over laparoscopy when performing lateral pelvic lymph node dissection (LPLND) with TME or extended TME (e‐TME) in locally advanced rectal cancer (LARC) is not known.

**Aims:**

This study was conducted to evaluate the outcomes of robotic and laparoscopic LPLND in patients with lateral pelvic node‐positive LARC with reference to intraoperative safety, postoperative morbidity, pathological indices including nodal yield and node positivity rates, lateral pelvic recurrence rates, and short term event‐free and overall survival.

**Methods and Results:**

In this retrospective single‐center study, consecutive patients with non‐metastatic histologically proven LARC and clinically significant lateral pelvic lymphadenopathy who had undergone laparoscopic or robotic LPLND with TME or e‐TME between 2014 and 2023 were included, all procedures having been performed by minimal‐access colorectal surgeons who were beyond the learning curve for either surgical approach. Of the 115 patients evaluated, 98.3% received neoadjuvant chemoradiotherapy, following which 27 (23.5%) underwent robotic and 88 (76.5%) laparoscopic LPLND with TME or e‐TME. The baseline clinicodemographic features, treatment‐related characteristics, and proportion of patients undergoing extended resections for persistent circumferential resection margin‐positive rectal cancer (22.7% vs. 18.5%, respectively) were statistically similar in both groups. When comparing robotic with laparoscopic resections, no significant difference was observed in intraoperative parameters including procedure‐associated blood loss (median 250 mL vs. 400 mL) and on‐table adverse events or conversion rates (none in either group), postoperative outcomes comprising clinically significant early (14.8% vs. 9.1%), intermediate (5.3% vs. 1.9%) and late (5.3% vs. 2.0%) surgical morbidity, re‐exploration rates (7.4% vs. 3.4%) and duration of hospital stay (median 6 days in both groups), or the pathological quality indices of margin involvement (7.4% vs. 2.3%), nodal yield (median 4 vs. 7 nodes) and lateral node positivity (22.2% vs. 26.1%), respectively. At a median 11 months follow‐up, oncological outcomes in terms of lateral pelvic recurrence rates (3.7% vs. 4.5%), 2‐year event‐free survival (78.7% vs. 79.3%) and 2‐year overall survival (83.1% vs. 93.8%) were also comparable.

**Conclusion:**

Surgical competence in laparoscopy may offset the potential benefits extended by robotic platforms. In a high‐volume setup with experienced minimal‐access surgeons, the clinical, pathological, and short‐term oncological outcomes associated with both approaches may be considered equivalent.

## 1 Introduction

Lateral pelvic lymph node (LPLN) metastasis has been reported to occur in up to 25% of patients with primary adenocarcinoma of the rectum, and is considered the most common cause for locoregional recurrence following curative‐intent rectal cancer surgery [1, 2, 3, 4]. Although extramesorectal nodal involvement was previously regarded as systemic disease, contemporary studies have reported superior rates of local control and overall survival by surgically addressing the lateral pelvic nodal basins with lateral pelvic lymph node dissection (LPLND), either at presentation or when lymphadenopathy persists after neoadjuvant therapy, when performing total mesorectal excision (TME) for the rectal primary [5, 6]. However, the procedure has been associated with greater intraoperative blood loss, longer operative duration, and higher complication rates, including functional deficits and genitourinary morbidity primarily attributed to the complex neurovascular anatomy of the pelvic sidewall [7, 8, 9].

Although the detrimental impact of positive lateral nodes on disease control is widely recognized, the technical challenges and adverse postoperative outcomes associated with LPLND have led to divergent strategies for the management of clinically significant lymphadenopathy across different centers around the world [10]. Presently, most patients with LPLN‐positive rectal cancer in North America and Europe are offered neoadjuvant chemoradiotherapy, with “selective” LPLND being considered for clinically persistent nodal disease, whereas East Asian guidelines additionally recommend LPLND for patients with T3–4 lower rectal cancer arising below the peritoneal reflection regardless of node‐positivity [11, 12].

In attempts to reduce postoperative procedure‐related morbidity, the surgical approach to lateral pelvic lymphadenectomy has also witnessed a transition from the conventional open access advocated for patients with locally advanced rectal cancer (LARC) to a minimally invasive approach, extrapolating the improved patient‐related outcomes reported with the latter in patients with organ‐confined rectal cancer undergoing TME [8, 13]. Recent studies have established the safety and oncological equivalence of laparoscopic surgery in addressing clinically significant lateral pelvic lymphadenopathy even in patients who have received neoadjuvant chemoradiotherapy [8, 14, 15, 16, 17]. Although the approach has a steep learning curve with the point of inflection reported to occur at approximately the 20th case for colorectal surgeons experienced in laparoscopy, outcomes have been reported to improve with experience [18].

Since its induction into surgical practice, robotic platforms have extended surgeons the functional dexterity and three‐dimensional perception associated with open surgery, along with superior ergonomics and a shorter learning curve, in addition to all the patient‐centric perioperative benefits of laparoscopy, serving to bridge the divide between the two approaches. Prospective data comparing laparoscopic and robotic TME for localized rectal cancer suggest that the latter is associated with superior intraoperative outcomes, a favorable morbidity profile facilitating faster recovery, and better oncological indices in terms of nodal yield and margin positivity [13]. Although studies have attempted to characterize and compare the morbidity profile of LPLND performed by each of these approaches to ascertain whether robotic platforms offer any additional advantage over laparoscopy, heterogeneity in inclusion criteria and receipt of neoadjuvant therapy, limited sample sizes of the comparator arms, and the potential for operator dependent selection biases when determining the surgical approach for lymphadenectomy are significant limitations precluding the wider applicability of the findings [19, 20, 21, 22].

This study was conducted to evaluate the outcomes of robotic and laparoscopic LPLND, undertaken either upfront or following chemoradiotherapy in patients with LARC and significant lateral pelvic lymphadenopathy, in terms of intraoperative safety, clinically significant postoperative morbidity, nodal yield, lateral pelvic recurrence rate, and survival.

## 2 Materials and Methods

### 2.1 Study Design

This cohort study was conducted in the Colorectal Division of the Department of Surgical Oncology at Tata Memorial Hospital, a tertiary cancer center in India, using prospectively collected data of patients treated between April 2014 and March 2023. Consecutive patients with non‐metastatic, histopathologically proven rectal adenocarcinoma arising distal to the sigmoid take‐off point on imaging and significant lateral pelvic lymphadenopathy, having undergone elective curative‐intent minimally invasive (laparoscopic or robotic) TME or extended TME (involving the partial resection of adjacent clinically‐involved anatomical structures and/or organs) with LPLND, regardless of the receipt of neoadjuvant therapy, were selected [23]. Primaries of the upper rectum with suspicious lateral pelvic lymphadenopathy were also included despite a predominantly cephalad lymphatic drainage pattern, as a significant proportion of these patients have been reported to harbor nodal metastases [24]. Patients with a history of having undergone pelvic surgery in the past, or presenting with locally advanced tumors requiring adjacent organ resections or pelvic exenterations (beyond‐TME procedures) were excluded, as were those with histologies other than adenocarcinoma.

### 2.2 Preoperative Assessment and Neoadjuvant Therapy

Following the standard assessment for rectal cancer, including a detailed history and physical examination, tumor markers, colonoscopy and biopsy, MRI pelvis, and contrast‐enhanced computerized tomography (CECT) of the thorax and abdomen, all patients were managed as per the decision of the multidisciplinary tumor board. On imaging, as reviewed by two radiologists, clinically significant LPLNs (inclusive of the obturator, and the internal, external and common iliac groups) were defined as morphologically suspicious nodes of rounded shape, ill‐defined borders, or heterogeneous signal intensities and size ≥ 7 mm (in the short‐axis) on the index pre‐neoadjuvant therapy scan, or post‐therapy size ≥ 4 mm (for internal iliac nodes) and ≥ 6 mm (for the other nodal groups) [11]. Corresponding to clinical stage III disease [25] and regarded as locally advanced cancer, these patients were considered for neoadjuvant therapy. Conventional long‐course RT with oral fluoropyrimidine‐based chemotherapy (LCCRT) was the preferred approach, the radiation dose ranging from 45–50.4 Gy in 25–28 fractions at 1.8–2 Gy/fraction per day, 5 days weekly. A transition towards short‐course RT (SCRT), dosed at 25 Gy in 5 fractions over 5 days, with consolidation chemotherapy and delayed surgery was observed in patients with locally advanced primaries (cT4a/b) and/or significant nodal burden (cN2) during the COVID‐19 pandemic, also taking into consideration emerging evidence at the time [26, 27]. The radiation field included the gross tumor volume, comprising the primary tumor with its local extensions and all clinically involved nodes including those in the lateral pelvic nodal basins, and the clinical target volume covering the draining locoregional nodes at risk (mesorectal, presacral, internal iliac and obturator nodes). RT boost was given at the discretion of the treating physician, the technique of delivery being either sequential or as simultaneous integrated boost (SIB), the latter allowing the simultaneous delivery of different levels of radiation dosing to different target volumes within a single treatment fraction.

### 2.3 Surgical Management

**FIGURE 1:**
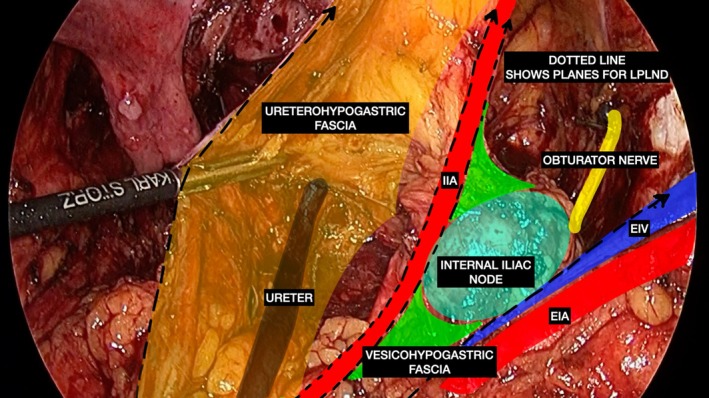
Endoanatomy of the pelvic sidewall following laparoscopic right lateral pelvic lymph node dissection depicting the surgical template of lymphadenectomy (dotted lines), with the ureterohypogastric fascia (orange) and the external iliac artery and vein (EIA and EIV) representing the medial and lateral limits of dissection, respectively. The vesicohypogastric fascia (green) along the internal iliac artery (IIA) divides this space into medial internal iliac and lateral obturator compartments.

Definitive surgery was performed at least 8 weeks after the completion of radiotherapy, with the type of resection, approach, and extent of lymphadenectomy decided by a panel of surgeons taking into consideration the distance of the primary from the anal verge, sphincter‐complex involvement, and the response to therapy on imaging [11]. TME or extended‐TME was performed with LPLND on the side(s) affected, the template (Figure 1) and technique of which have been standardized and described previously [28]. Unless involved by nodal metastases, care was taken to preserve the autonomic nerve plexus and the pelvic vasculature. Intraoperative adverse events and any consequential need to convert the procedure to open (defined as a laparotomy made to complete any part of the surgical resection, exclusive of incisions made to facilitate the anastomosis or for specimen extraction) were recorded. All procedures were performed either laparoscopically or by robotics, using the da Vinci Xi Surgical System (Intuitive Surgical, Sunnyvale, CA). The two colorectal cancer surgeons involved in the study (AS and AD) had been extensively trained in laparoscopic and robotic rectal surgery, having individually undertaken over 500 such procedures and over 50 lateral pelvic lymphadenectomies (for rectal, urological and gynecological malignancies) before the study period and performing between 100 and 200 minimal‐access colorectal cancer surgeries every year [18].

### 2.4 Postoperative Outcomes

Postoperatively, the Clavien–Dindo classification of surgical complications was used, with grade III or greater morbidity considered clinically significant [29]. These were further subclassified into early (occurring within 30 days of surgery), intermediate (from 31 to 90 days) and late complications (beyond 90 days postoperatively). Patients were followed up 3‐monthly during the first 2 years, every 6 months up to 5 years, and annually thereafter. Serum CEA was recorded at each visit. CECT of the thorax, abdomen and pelvis was performed 1 year following surgery and annually thereafter. Colonoscopy, when complete preoperatively, was performed at 1‐, 3‐, and 5‐yearly intervals. Recurrences when detected were classified as locoregional (for disease confined to the pelvis) or systemic (inclusive of visceral, peritoneal and extrapelvic nodal metastases). Event‐free survival (EFS) was considered as the time interval between the date of surgery and the date of occurrence of an event defined either as tumor recurrence or death from any cause, or in the absence of events, the date of last follow‐up. Overall survival (OS) was considered as the time period between the date of surgery and the date of death from any cause or date of last contact. The data was censored on September 30, 2023.

### 2.5 Statistics

Statistical analysis was performed using SPSS Statistics for Windows Version 23.0 (IBM Corp, Armonk, NY, USA). Continuous variables were expressed either as mean and standard deviation or median and interquartile range, and compared using the independent Student's t‐test (for two groups) or the one‐way ANOVA (for more than two groups). Categorical data were presented as counts and percentages and the Chi‐square test used to identify associations between these variables. When the expected cell count was less than 5, Fisher's exact test (two‐sided) was performed. EFS and OS were calculated, and group‐specific survival outcomes were analysed by plotting Kaplan–Meier curves, with the comparisons made using the log‐rank test. For all parameters, a *p* value < 0.05 was considered statistically significant.

### 2.6 Ethics Statement

This study involved the analysis of data obtained for clinical purposes, with informed written consent having been obtained from all patients prior to any treatment or intervention. The study was approved by the Institutional Ethics Committee (Project No. 4570, Ref No. OIEC/4570/2024/00002 dated November 11, 2024) and conducted in accordance with the ethical standards of the Helsinki Declaration of 1975 and its subsequent amendments.

The research protocol complied with the Strengthening the Reporting of Observational Studies in Epidemiology (STROBE) guidelines.

## 3 Results

**FIGURE 2:**
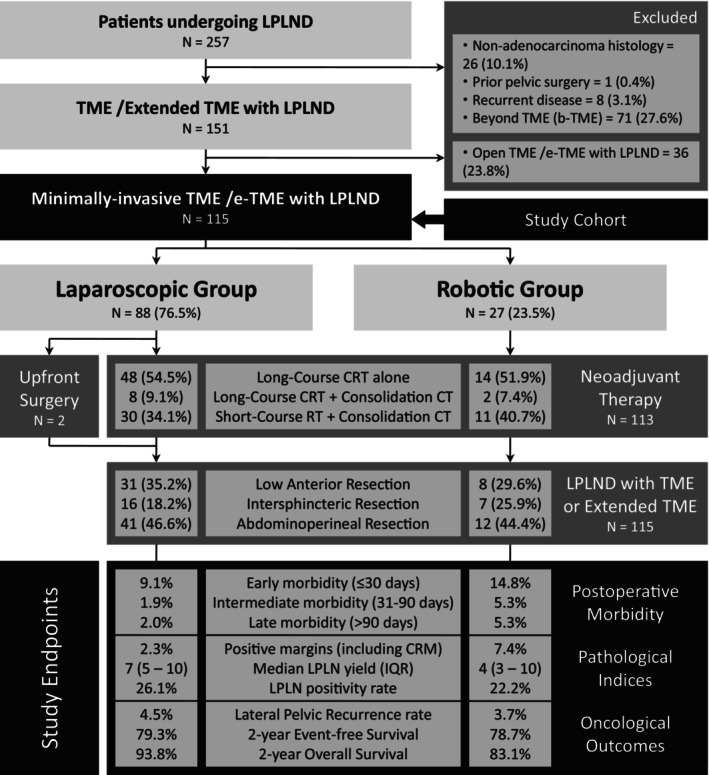
Flow diagram illustrating the study sample inclusion and exclusion criteria with the surgical treatment offered and outcomes of patients undergoing laparoscopic and robotic total mesorectal excision (TME) or extended TME with LPLND (*n* = 115).

During the study period, 257 patients undergoing LPLND were identified, of which 115 patients satisfying the inclusion criteria were selected for analysis (Figure 2).

### 3.1 Patient Characteristics

Overall, 98.3% of patients received neoadjuvant therapy, of which 21.2% also received a radiation boost to involved pelvic nodes with or without inclusion of the primary. Two patients with early‐stage upper rectal primaries and clinically insignificant mesorectal nodes but with suspicious LPLNs underwent upfront laparoscopic low anterior resection and unilateral LPLND. For the remaining patients, following a period ranging from 8 to 12 weeks post‐radiotherapy, minimally invasive resection of the primary with selective LPLND was performed, 27 (23.5%) of which were robotic and 88 (76.5%) laparoscopic. The baseline clinicodemographic features, tumor‐related characteristics, and treatment modalities offered, including the proportion undergoing extended‐TME and the laterality of LPLND, were comparable between patients in both groups (Table 1).

**TABLE 1:** Baseline clinicodemographic features, treatment modalities offered and tumor‐related characteristics of patients included of the study cohort (*n* = 115).

			*n*	Sum total/mean	Laparoscopic group (*n* = 88) 76.5%	Robotic Group (*n* = 27) 23.5%	*p*
Age (mean ± SD), in years			115	44.5 ± 12.24	43.9 ± 11.48	46.5 ± 14.50	0.330
Gender	Male		72	62.6%	52	20	0.180
	Female		43	37.4%	36	7	
ECOG Score	0		6	5.2%	5	1	0.630
	1		104	90.4%	78	26	
	2		5	4.4%	5	0	
Body mass index (mean ± SD), in kg/m^2^			115	23.5 ± 3.94	23.7 ± 3.67	22.7 ± 4.72	0.272
Distance from anal verge (median, IQR), in cm			115	3 (1–5)	3 (2–5)	2 (1–5)	0.366
Baseline serum CEA (median, IQR), in ng/ml			67	5.4 (2.1–11.5)	6.1 (2.2–11.1)	4.3 (1.9–11.8)	0.663
Neoadjuvant therapy	Yes	LCCRT alone	62	53.9%	48	14	1.000
		LCCRT + consolidation CT	10	8.7%	8	2	
		SCRT + consolidation CT	41	35.7%	30	11	
	No		2	1.7%	2	0	
Boost	No		89	78.8%	70	19	0.385
	Yes	Simultaneous integrated boost	13	11.5%	9	4	
		Sequential boost	11	9.7%	7	4	
Surgery	Low anterior resection		39	33.9%	31	8	0.645
	Intersphincteric resection		23	20.0%	16	7	
	Abdominoperineal resection		53	46.1%	41	12	
Extended resections	Conventional TME		90	78.3%	68	22	0.792
	Extended TME		25	21.7%	20	5	
Blood loss (median, IQR), in ml			115	400 (200–600)	400 (200–600)	250 (200–400)	0.074
Re‐exploration	Yes		5	4.3%	3	2	0.335
	No		110	95.7%	85	25	
Hospital stay (median, IQR), in days			115	6 (5–8)	6 (5–8)	6 (5–8)	0.311
Margin status^a^	Negative		111	96.5%	86	25	0.234
	Positive		4	3.5%	2	2	
Pathological T stage (pT)	pT0		31	27.0%	24	7	0.804
	pT1		5	4.3%	4	1	
	pT2		19	16.5%	14	5	
	pT3		54	47.0%	40	14	
	pT4		6	5.2%	6	0	
Pathological N status (pN)	pN0		57	49.6%	43	14	0.872
	pN1		40	34.8%	30	10	
	pN2		18	15.7%	15	3	
LPLND Side	Right		42	36.5%	34	8	0.284
	Left		49	42.6%	34	15	
	Bilateral		24	20.9%	20	4	
LPLN yield (median, range)	Right		42	6 (1–17)	6 (1–17)	7 (3–16)	0.192
	Left		49	6 (0–25)	6.5 (0–25)	3 (0–12)	
	Bilateral		24	10 (4–28)	10 (4–28)	9.5 (7–19)	
Pathological LPLN status	Negative		86	74.8%	65	21	0.803
	Positive		29	25.2%	23	6	
Lateral nodal positivity rate			115	25.2%	26.1%	22.2%	
Follow up (median, IQR), in months			109	11 (6–22)	10 (6–20.5)	12 (7–51.5)	0.027

Abbreviations: ECOG, Eastern Cooperative Oncology Group scoring; IQR, Interquartile range; LCCRT, long course chemoradiotherapy; LPLND, lateral pelvic lymph node dissection; LPLN, lateral pelvic lymph node; SD, standard deviation.

^a^ Including the circumferential resection margin.

### 3.2 Postoperative Outcomes

In the immediate postoperative period, four patients (two each in either group) required re‐exploration for conditions ranging from distal margin positivity and fascial dehiscence with evisceration to acute intestinal obstruction (secondary to empty‐pelvis syndrome and port‐site hernia) at a median of 10 days following surgery (range 6–21 days).

The pathological indices (including stage, median LPLND yield and positivity rate) of subjects in both arms of the study were comparable, with 29 patients (25.2%) of the overall population harboring positive LPLNs, of which 11 (37.9%) were bilateral. Clinically significant Clavien‐Dindo grade III or greater morbidity was observed in 13.9% of the cohort, the incidence being proportionate in both study groups (Table 2). Among these, pelvic collections requiring drainage comprised the single most common complication seen, with 18.2% of those detected complicated by superadded infection or impinging on adjacent anatomical structures causing symptoms. Surgical site occurrences (including seromas, superficial surgical site infections, and wound dehiscence) were exclusively seen during the first month after surgery, representing the most common complication in this period.

**TABLE 2:** Postoperative complications and Clavien Dindo grading in the early (up to 30 days after surgery), intermediate (31–90 days) and late (beyond 90 days) post‐operative period.

Morbidity		*n*	Overall	Laparoscopic (*n* = 88)	Robotic (*n* = 27)	*p*
Time	Clavien–Dindo grade					
Early (≤ 30 days) *n* = 115	Grade 0	75	65.2%	58	17	0.472
	Grade I–II (clinically minor)	30	26.1%	23	7	
	Surgical site occurrences^a^	12		10	2	
	Pelvic collections^b^	8		4	4	
	Stomal complications^c^	5		4	1	
	Bowel‐related^d^	4		4	0	
	vDVT	2		1	1	
	Urinary^e^	1		1	0	
	Grade III–V (Clinically significant)	12	10.4%	8	4	
	Surgical site occurrences	5		3	2	
	Bowel‐related	4		3	1	
	Stoma related	1		1	0	
	Pelvic collections	1		0	1	
	Urinary	1		1	0	
	Sepsis with AKI	1		1	0	
Intermediate (31–90 days) *n* = 72	Grade 0	59	81.9%	44	18	0.445
	Grade I–II (clinically minor)	11	15.3%	11	0	
	Pelvic collections	6		6	0	
	Urinary	5		5	0	
	DVT	1		1	0	
	Grade III‐V (Clinically significant)	2	2.8%	1	1	
	Pelvic collections	1		0	1	
	Mortality	1		1	0	
Late (> 90 days) *n* = 70	Grade 0	63	90.0%	45	0	0.472
	Grade I–II (clinically minor)	5	7.1%	5	0	
	Pelvic collections	4		4	0	
	Urinary	1		1	0	
	Grade III–V (clinically significant)	2	2.9%	1	1	
	Pelvic collections	2		1	1	

^a^ Surgical site occurrences included seromas, surgical site infections, and wound dehiscence.

^b^ Pelvic collections were radiologically documented sequestrations of fluid in the pelvic cavity with or without associated locoregional lymphedema or mass effect caused by impingement on adjacent anatomical structures.

^c^ Stoma‐related complications comprised stomal prolapse or retraction, necrosis, and parastomal hernia.

^d^ Bowel‐related morbidity inclusive of ileus, obstruction, and anastomotic dehiscence resulting in leak.

^e^ Urinary complications comprised urinary retention requiring catheterization (including clean intermittent catheterization), incontinence, ureteral strictures, hydroureteronephrosis, urosepsis, or renal failure.

**FIGURE 3:**
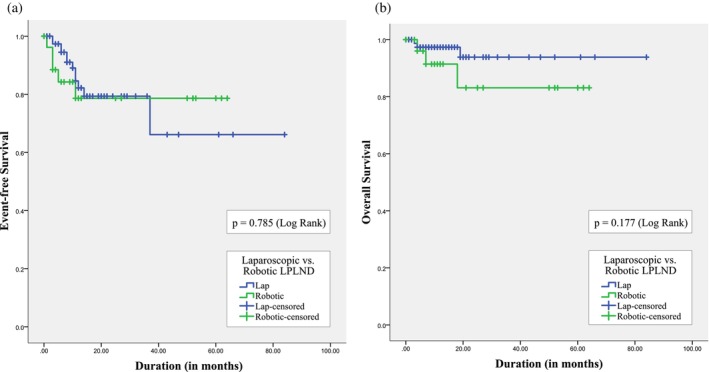
Kaplan–Meier estimates for (a) event‐free survival and (b) overall survival of patients in the laparoscopic and robotic lateral pelvic lymph node groups.

During the median 11‐month follow‐up period (IQR 6–22 months), 17 patients developed recurrences at a median of 8 months (IQR 4–11 months) post‐surgery, of which 15 were systemic. (Table 3) Overall, lateral pelvic recurrences were noted in five patients, comprising 4.5% and 3.7% of subjects in the laparoscopic and robotic groups, respectively. At the time of censoring, 96 patients of the study cohort were alive and disease‐free, and six had died, five of whom had succumbed to recurrent cancer. The 2‐year EFS and OS of this cohort with LARC were 79.3% and 91%, respectively. No significant difference was noted in group‐specific comparisons of these parameters (2‐year EFS of 79.3% and 78.7%; 2‐year OS of 93.8% and 83.1% for patients in the laparoscopic and robotic arms, respectively) (Figure 3).

**TABLE 3:** Anatomical distribution of recurrences observed in the study population during the follow‐up period.

			*n*	Laparoscopic (*n* = 88)	Robotic (*n* = 27)	*p*
No relapse			98	76	22	0.543
Recurrence	Overall		17	12	5	
	Distant	Overall	15	10	5	
		Visceral^a^	9	7	2	
		Peritoneal	6	5	1	
		Extrapelvic nodes^b^	6	3	3	
		Skeletal	2	1	1	
	Locoregional	Overall	6	5	1	
		Anastomotic	1	1	0	
		Lateral pelvic	5	4	1	
		Presacral	2	2	0	
Lateral pelvic recurrence rate			5	4.5%	3.7%	1.000

^a^ Inclusive of metastases to the liver, adrenals, lungs, or brain.

^b^ Comprising the retroperitoneal, mediastinal, or cervical nodal groups.

## 4 Discussion

In this study conducted at a high‐volume center, we sought to determine whether robot‐assisted LPLND, when performed with TME or e‐TME in patients with LARC who were preoperatively downstaged with neoadjuvant therapy, extended a potential benefit when compared to a laparoscopic approach with regard to perioperative, pathological, and oncological outcomes. Our results revealed that despite robotic platforms facilitating surgical precision with features such as superior optics, enhanced degrees of freedom of movement, tremor‐filtering and motion‐scaling, there was no significant difference in either the intraoperative parameters (including on‐table adverse events, blood loss or conversion rates), or the surgical morbidity profile of subjects in the immediate, intermediate, and late postoperative period as suggested by the severity of complications observed, re‐exploration rate, and duration of hospital stay between the two minimal‐access approaches. Additionally, the pathological assessment revealed no difference in the oncological quality of resection, with respect to the rate of circumferential resection margin (CRM) positivity, distal margin involvement, nodal yield, and lateral node positivity rate despite a comparable number of e‐TMEs and intersphincteric resections having been performed in both groups. The incidence of urinary morbidity in the postoperative period was also similar.

Compared with laparoscopy, robotic resections have been associated with superior perioperative and functional outcomes in CRM‐negative localized rectal cancers, primarily attributed to the enhanced precision facilitated by a platform that offers magnified, three‐dimensional vision with wrist‐like dexterity and maneuverability via a seamless interface [13]. However, the evidence for either approach in LARC is not robust, with tumor extension beyond the TME plane having been historically considered a contraindication to minimally invasive resections, and CRM‐threatened tumors having been selectively excluded both from trials designed to establish the non‐inferiority of laparoscopic over open resections [30, 31, 32] and those conducted to evaluate the outcomes of robotic surgery [13]. Nevertheless, data from high‐volume centers suggest that such patients may benefit from this approach in terms of a favorable morbidity profile and shorter length of hospital stay without compromising oncological outcomes or survival compared with the conventional open surgery [33, 34]. Further analyses of the minimal‐access techniques have found that robotic surgery may offer a benefit over laparoscopy in terms of conversion rates and time‐to‐discharge at the cost of theater time [35]. Experience and surgical competence have been associated with these results since, with increasing experience, surgeons substitute visual cues for tactile sensation, avoiding excessive force or traction while manipulating tissues.

Similarly, minimally invasive LPLND in LARC has also been associated with significantly better perioperative morbidity and comparable oncological outcomes [14, 36]. Although evidence for the safety and technical feasibility of robotic LPLND over laparoscopic dissection is derived from small retrospective studies, very few have compared the two approaches in patients post chemoradiotherapy [19, 20, 22]. In our cohort, nearly all patients received neoadjuvant radiation, with a significant proportion (35.7%) presenting with locally advanced, high‐risk tumors considered for SCRT and consolidation chemotherapy [26, 27]. An additional boost to the LPLNs with or without inclusion of the primary was also considered based on evidence suggesting better locoregional control [37, 38].

A technically challenging procedure in itself, the receipt of neoadjuvant radiation has been known to add to the surgical complexity of LPLND, with tissue friability and interstitial edema manifesting early in the post‐radiotherapy period, later progressing to fibrosis with increasing intervals to surgical resection, leading to greater difficulty in the identification and delineation of anatomical planes, and consequently, greater intraoperative blood loss, longer operative duration, and higher conversion rates. Robotics may potentially offer significant benefits in this scenario, providing a stable platform with stereoscopic vision and magnification facilitating the visualization and preservation of the pelvic autonomic nerve fibers and hence genitourinary function, superior control around the pelvic vasculature, and the provision for intracorporeal suturing. Moreover, features such as haptic zoom minimize the excessive fogging associated with extensive tissue exudation when operating within the confines of the pelvis, reducing operator and assistant fatigue. Unlike laparoscopy, accessibility to the lateral pelvic sidewall is not compromised by a reduction in the degrees of range of movement or the presence of adverse patient factors including obese body habitus or narrow pelvic inlet dimensions, both of which would otherwise have predisposed to a difficult nodal dissection. In this study, however, these advantages failed to translate into superior perioperative outcomes. As a metric for surgical quality and a predictor of postoperative recovery, a lower median blood loss was recorded with robotic surgery in the present study. However, this failed to reach statistical significance, contrary to findings from recent literature wherein robot‐assisted LPLND was associated with reduced blood loss when compared to a laparoscopic approach [19, 22].

In the interpretation of our findings, it is important to note that all procedures were performed by experienced minimal‐access surgeons who were beyond the learning curve for both approaches, a factor that has been associated with surgical outcomes [18, 39]. The number of cases reported necessary to acquire working proficiency in laparoscopic colorectal surgery ranges from 20 to 70 [40]. However, the attributes of robotic systems may facilitate an easier transition from open access to a minimally invasive approach, with the learning curve for robotic‐assisted resections for surgeons competent in laparoscopy estimated to range from 15 to 25 cases [41]. Moreover, outcomes with robot‐assisted resections have been reported to be independent of variables traditionally considered essential when negotiating the laparoscopic learning curve, including years of surgical experience, work volumes, size of the hospital facility, and teaching status [42]. In practice, where experience and competence vary widely between surgeons, robotic platforms may serve to eliminate these differences, delivering results that are independent of volumes or operator experience, and enabling surgeons in the early phases of their learning curves to achieve results that are comparable with their more experienced colleagues [39].

With the caveat of a limited follow‐up, no difference was observed in the incidence of lateral recurrences for either group in the cohort, with EFS and OS of patients undergoing TME and LPLND comparable with data from contemporary studies [4, 5, 15]. Nonetheless, this study evaluated outcomes associated with the minimal access surgical approaches for LPLND performed with TME or extended‐TME at a single institution in a homogeneous cohort of patients with locally advanced rectal cancer and clinically significant lateral pelvic lymphadenopathy. Apart from the stringent eligibility criteria including only patients with either mesorectum‐confined rectal cancer or marginal extramesorectal extension not requiring multivisceral resections, nearly all subjects in the cohort received neoadjuvant chemoradiotherapy, and the procedures were performed by surgeons experienced in both techniques using a uniform standardized lymphadenectomy template. To ensure that the observed outcomes could be attributed to LPLND, MRF‐positive, clinical T4 tumors requiring beyond TME resections were excluded as the morbidity profile of these surgeries could affect postoperative recovery and hence the study endpoints. In contrast to previous studies that have compared outcomes of the minimal‐access approaches in patients with CRM‐negative rectal cancer undergoing TME with LPLND, the inclusion of patients with high‐risk LARC (comprising cT4 and/or cN2 disease with persistent CRM involvement post‐chemoradiotherapy) undergoing minimally invasive extended resections allowed for the comparison of outcomes associated with both TME and e‐TME with LPLND, hence representing a real‐world scenario.

### 4.1 Limitations

The disproportionate number of subjects in each of the study groups was reflective of the limited availability of robotic slots at the Institute, despite the indications for either approach remaining comparable. Additionally, when compared with laparoscopy, the robotic approach for LPLND was introduced later in the study period at our center, partly accounting for the smaller size of this arm. Despite this disparity, the two groups were evenly matched with respect to the baseline clinicodemographic features, the receipt of neoadjuvant therapy, surgical procedure, and proportion of extended resections. Although studies comparing the two minimally invasive operative approaches have reported superior nodal yield with robotic surgery by facilitating access to the distal aspect of the internal iliac region in particular, this was not specifically evaluated in the present study, the nodal yield in both groups being comparable [19, 22]. We did not assess operative duration as a comparative parameter as it was not uniformly recorded in our database. Although robotic resections take longer to perform than laparoscopy on account of robotic setup and docking, it is seldom feasible to isolate LPLND time from the total duration of surgery, making a direct comparison between the approaches difficult. Secondly, the duration of surgery may also be considered a function of both rectal resection and LPLND, further complicating the breakdown of theater time unless data is collected in a prospective setting. With regard to postoperative morbidity, literature favors the robotic approach over laparoscopy when comparing rates of urinary retention and genitourinary function [19]. However, sexual dysfunction was not evaluated in this study. Also, the median follow‐up period was short, hindering our efforts to identify procedure‐associated long‐term complications and rates of disease relapse.

Lastly, this study, conducted at a high‐volume tertiary cancer center, involved experienced minimal access surgeons and a standardized dissection template, minimizing operator‐related and procedure‐specific heterogeneity. In this regard, the extrapolation of data and generalizability of results may be limited for operators with varying degrees of surgical competence.

## 5 Conclusions

In a study cohort that included extended rectal resections, robotic TME or e‐TME with LPLND was found to be equivalent to the laparoscopic approach with regard to safety, postoperative morbidity, pathological quality indices, and short‐term survival outcomes. Although robotic platforms facilitate superior operative dexterity and precision by virtue of advanced instrumentation and enhanced optics, these benefits may be offset by surgical competence in laparoscopy. In a high‐volume setup with experienced minimal‐access surgeons, this approach may not offer a significant advantage over laparoscopic surgery.

## Author Contributions

**Joseph Mathew:** conceptualization (equal), data curation (equal), formal analysis (equal), investigation (equal), methodology (equal), resources (equal), software (equal), validation (equal), visualization (equal), writing – original draft (equal), writing – review and editing (equal). **Yogesh Kisan Bansod:** data curation (equal), visualization (equal), writing – original draft (equal). **Nishant Yadav:** conceptualization (equal), methodology (equal). **Janesh Murugan:** conceptualization (equal), data curation (equal). **Kovvuru Bhaskar Reddy:** data curation (equal), writing – original draft (equal). **Mufaddal Kazi:** conceptualization (equal), formal analysis (equal), investigation (equal), methodology (equal), resources (equal), validation (equal), writing – review and editing (equal). **Ashwin DeSouza:** conceptualization (equal), resources (equal), supervision (equal), validation (equal), writing – review and editing (equal). **Avanish Saklani:** conceptualization (equal), project administration (equal), resources (equal), supervision (equal), validation (equal), writing – review and editing (equal).

## Ethics Statement

The study was approved by the Institutional Ethics Committee (Project No. 4570, Ref No OIEC/4570/2024/00002 dated November 11, 2024) and conducted in accordance with the ethical standards of the Helsinki Declaration of 1975 and its subsequent amendments.

## Consent

Individual consent was taken as part of treatment.

## Conflicts of Interest

The authors declare no conflicts of interest.

## Data Availability

The data that support the findings of this study are available from the corresponding author upon reasonable request.
